# A Case Study On Anticipated End-of-Life Caregiving Among The Millennial American Born Chinese

**DOI:** 10.1093/geroni/igab046.2843

**Published:** 2021-12-17

**Authors:** Tongtong Li, Aileen Zhang, Ruotong Liu, Iris Chi

**Affiliations:** 1 University of Southern California, Arcadia, California, United States; 2 University of Southern California, Los Angeles, California, United States; 3 University of Southern California, University of Southern California, California, United States

## Abstract

Millennial American Born Chinese (ABCs) are in a double jeopardy position with end-of-life (EOL) care for their immigrant parents, because of both cultural and generational clashes. There is no existing empirical study about the millennial ABCs’ attitudes or behaviors towards EOL caregiving. Our study is the first one to explore the millennial ABCs’ anticipated EOL caregiving behaviors, support and resources needed, attitudes towards terminal illness disclosure and advance care planning (ACP) discussion with their parents, and how acculturation influences. A qualitative in-depth phone interview using a case study approach, with a scenario of caring for parents with Parkinson’s disease and stage IV lung cancer, was adopted. Participants were recruited via convenience sampling, and a total of 27 (18 females and 9 males with an average age of 25) passed the screening and completed the interviews. Using the directed content analysis, researchers identified two themes: EOL caregiving and EOL decision making, which included five sub-themes: caregiving behaviors, needed supports and resources, care arrangement decision, terminal illness disclosure, and ACP. Both traditional Chinese culture of familism and filial piety, and western culture of autonomy and patients’ rights to know were exhibited in every theme. Most participants did not fully understand ACP concept, but they were willing to initiate ACP conversation after comprehending ACP concept. This study constitutes an essential step towards understanding the millennial ABC EOL caregivers’ financial, physical, and emotional needs from family, community and government, better establishing corresponding policies, and promoting public education in ACP to benefit this minority group.

